# Synovialosarcoma of the pharynx: A case report and literatture review

**DOI:** 10.1016/j.ijscr.2021.02.025

**Published:** 2021-02-13

**Authors:** Chaker Kaoutar, Ahmed Brahim Ahmedou, Youssef Oukessou, Redallah Abada, Roubal Mohamed, Mahtar Mohamed

**Affiliations:** ENT, Face and Neck Surgery Department, IBN ROCHD University Hospital, Faculty of medicine and Pharmacy, Hassan II University, Casablanca, Morocco

**Keywords:** Synovial sarcoma, Pharynx, Immunohistochemistry, Treatement

## Abstract

**Introduction:**

Synovial sarcoma is a rare tumor to be encountered in the head and neck region and is always a challenge in terms of diagnosis, treatment, as our case.

**Presentation of case:**

We present a 23-year old female patient with synovial sarcoma of posterolateral pharyngeal wall. The radiological and clinicopathological features along with various diagnostic tests and treatment options are discussed.

**Discussion:**

The objective of this study is to describe - from a clinical case reported from our institution, and from literature review- the clinical, radiological and histological features of pharyngeal synovial sarcoma and to discuss its therapeutic management.

**Conclusion:**

Synovial sarcoma of pharynx is extremely a rare tumor in current practice.

## Highlights

• Synovial sarcoma is a rare malignant neoplasm.
• The location in pharynx is extremely rare.
• The diagnosis of synovial sarcoma is confirmed by surgical biopsy.
• The objective of this study is to describe the clinical, radiological and histological features of pharyngeal synovial sarcoma and to discuss its therapeutic management.

## 1 Introduction

This work has been reported in line with the SCARE 2020 criteria. [21]

Synovial sarcoma is a rare malignant neoplasm that arises from the primitive pluripotent mesenchymal cells near to or remote from the articular surfaces [1]. It commonly occurs in the extremities, followed by the head and neck region which accounts for up to 10 %, 7 %of all case [2].It occurs mainly in adolescents and young adults, has a slight male predominance [3].The first case of head and neck synovial sarcoma which occurred in the pharynx was described in 1954 by Jernstrom [2,4].Since then there have been several independent case reports. However, there are very few case series of synovial sarcoma of the pharynx in literature.

The objective of this study is to describe - from a clinical case reported from our institution, and fromliterature review- the clinical, radiological and histological features of pharyngeal synovial sarcoma and to discuss its therapeutic management.

## 2 Case report

A 23-year old female patient reported to the Department of Head and Neck surgery, with a chief complaint of cervical algia and left otalgia sinceone year. Patient also gave a history of mouth breathing, snoring, dysphonia and dysphagia associated with taking solid foods over the last 4 months. All evolving in a context of weight loss without been unencrypted.

**Fig. 1:**
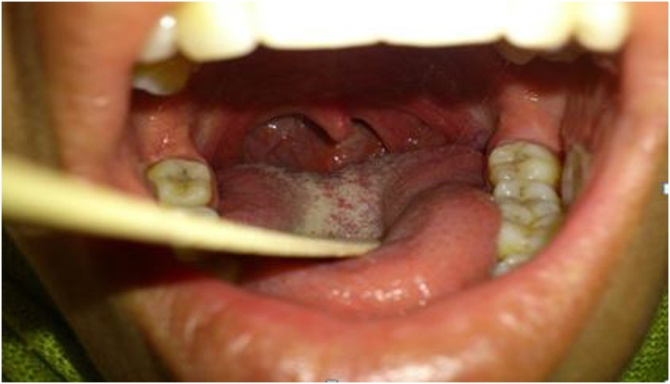
Clinical examination showed the circumscribed growth at the posterolateral wall of pharynx.

During initial clinical examination, on inspection, a huge circumscribed growth at the posterolateral wall of pharynx. The swelling was covered by normal mucosa (Fig. 1). The examination of cranial nerve was totally normal either the otoscopic examination.

Panendoscopy showed a regular bulge of the posterolateral wall of the oropharynx and the hypopharynx, approximate size 6*6 cm, without laryngeal involvement, covered by normal mucosa, and it was firm, well limited, and non tender on palpation.

**Fig. 2:**
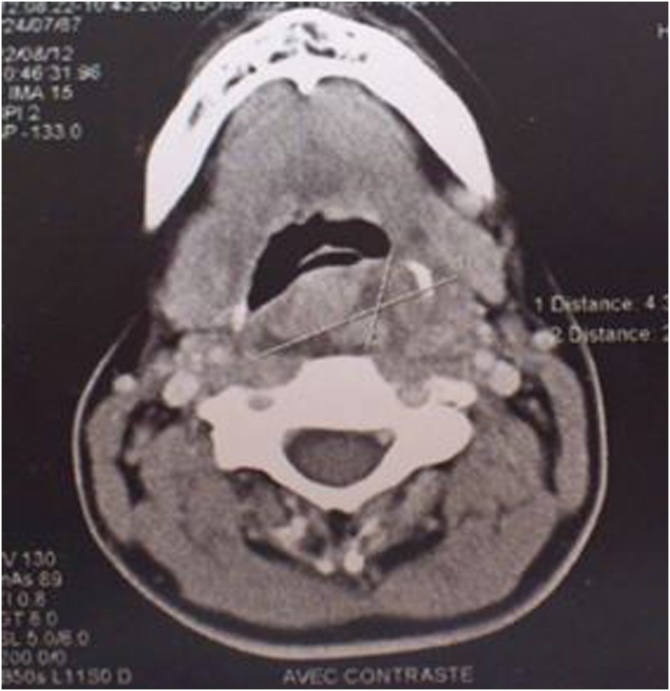
The scan revealed a heterogeneous mass of the left paropharyngeal space and retropharyngeal of the oropharynx.

The scan of face and neck revealed aheterogeneous mass of the left paropharyngeal space and retropharyngeal of the oropharynx6 cm wide, evoking a necrotic tumor (Fig. 2).

**Fig. 3:**
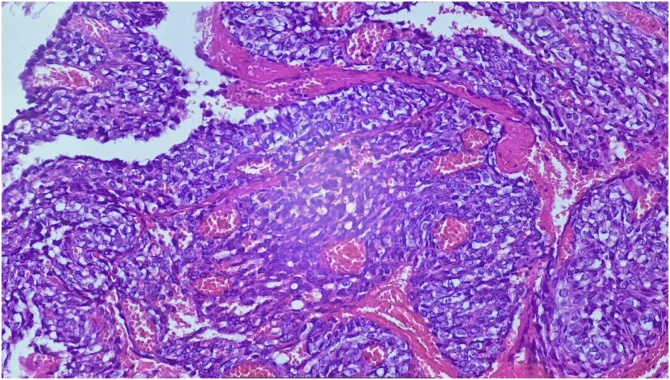
Showing a fusocellular mesenchymal proliferation.

**Fig. 4:**
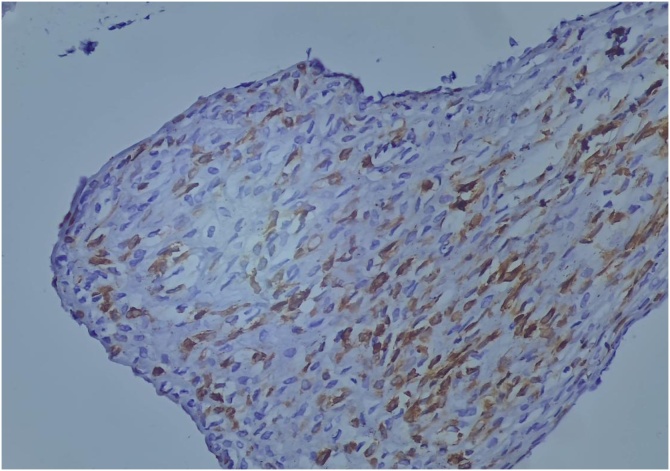
Immunohistochemistry showed a (anti-CD99+, anti bcI+, anti-vimentine+).

The biopsy after incision of the mucosa objectified a fusocellular mesenchymal proliferation (Fig. 3) on slide review additionally to IHC (immunohistochemistry) showed:(anti-CD99+, anti bcI+, anti-vimentine+, anti-PS100-, anti-cytokine-, anti-desmine-, anti CD34-) (Fig. 4), which suggested a synovialcell sarcoma or a spindle cell carcinoma.

Based on clinical examination, histopathological examination with IHC and radiological interpretation, the diagnosis of soft tissue sarcoma of the posterolateral pharyngeal wall was made.

The extension assessment includes a chest CT scan, abdominal ultrasound and bone scintigraphy, shows no evidence of any metastatic lesion.

The patient was put under chemotherapy while waiting for radiotherapy according to the decision of the multidisciplinary staff. The evolution was marked by the installation of typical respiratory symptomatology: dry cough, chest pain and dyspnea at rest, after the 2nd course of chemotherapy. The thoracic CT showed multiple metastatic lesions. So, the patient was put on palliative chemotherapy.

## 3 Discussion

Sarcomas of the head and neck are rare, accounting for only 1 % of all head and neck malignancies [5]. Synovial sarcomas (SS) comprise about 10 % of all soft tissue sarcomas, with SS of the head and neck (SS-HN) representing less than 0.1 % of all head and neck cancers [5,6]. Males are affected twice as often as females [7]. The first case of primary hypopharyngeal SS reported by Jernstorm in 1954 [[2], [3], [4]], since that several independent case reports have been published.

Synovial sarcoma is an intriguing entity owing to its rarity, controversial origin, challenging diagnosis and restricted treatment protocols [8]

It is frequently misdiagnosed as a benign lesion due to its smooth margins, an associated cystic component and lack of aggressive infiltration [9]. Ithas been postulated to arise from the malignant degeneration of pluripotential mesenchymal cells near or even remotefrom articular surfaces, tendons, tendon sheaths, juxta-articularmembranes, and facial aponeuroses [10,11].

The tumor usually occurs as an asymptomatic mass until it attains sufficient size to cause pressure effects on neighboring structures. This process is usually more rapid in head and neck Synovial sarcoma than those in the extremities [1]. Histopathologically, Synovial Sarcoma can be divided into 2 main subtypes, monophasic and biphasic. Monophasic SS is the most common subtype, composed of monomorphic spindle cells arranged in long, intersecting fascicles. Biphasic SS are characterized by welldeveloped glandular epithelial structures in addition to the spindle cell component [12].Synovial Sarcomamay also present as a poorly differentiated round cell sarcoma often arranged in pericytomatous pattern (poorly differentiated sarcoma). This is not a distinct subtype of SS; rather it represents a form of tumor progression that can occur in either monophasic or biphasic Synovial Sarcoma [2].On cytogenetic analysis typically harbours a t(X;18) (p11.2;q11.2) translocation with fusion between SSX1 and SYT genes with a biphasic appearancein two-thirds of cases whilst the remainder show a fusionbetween SSX2 and SYT genes [[13], [14], [15]].

Synovial sarcoma of head and neck has an aggressive nature and a guarded prognosis [8].A multidisciplinary approach has to be formulated to limit recurrences and prevent metastasis. There is no indication for prophylactic neck dissection except when palpable and enlarged lymph nodes arepresent [8]; Metastasis is mainly to the lungs, followed by lymph nodes and bone [13]. Most metastasis originate from haematogenous dissemination, although up to 20 % spread occur through the lymphatics to regional lymph node [16].

There was also no statistical significant difference when comparing the different therapy modalities used: surgery vs. surgery with radiotherapy alone vs. surgery with chemoradiotherapy [12]. Adjuvant radiotherapy is added to improve local tumor control if resection is inadequate [12]. Similar studies from Mayo clinic and MD Anderson showed no significant difference between different therapy modalities [17,18].

Local excision is followed by high recurrence rates (60–90 %), usually within 2 years. Daveau et al. study supports the use of adjuvant radiotherapy as it improved the overall survival rates with few recurrences in patients in whom the primary tumour was treated with surgical resection and radiotherapy [13,16]. Role of chemotherapy in the management of synovial sarcoma is still debatable. A meta-analysis of all sarcomas, including 10 % synovial sarcomas, found that doxorubicin chemotherapy significantly delayed local recurrence and remote metastasis and increased overall recurrence-free survival [13]. Other studies have shown benefits with ifosfamide based chemotherapy [15,19]. Literature presents with few cases of head and neck synovial sarcoma with no universal consensus for the role of chemotherapy but as the tumour size was more than 5 cm in our patient, chemotherapy was considered as a treatment option [8]. Overall, the 5-year survival rate in head and neck synovial sarcoma is around 40–60 % [13,15]. Decreased survival rates are seen with large tumour size (greater than 5 cm), positive margin status, and high tumour grade (Grade 3) [20].

## 4 Conclusion

Synovial sarcoma of pharynx is extremely a rare tumor in current practice, it is also a difficult tumor to be diagnosed beside that Literature presents with few cases with no universal consensus for treatment which makes the challenge.

## Conflicts of interest

The authors declare having no conflicts of interest for this article.

## Funding

None.

## Ethical approval

I declare on my honor that the ethical approval has been exempted by my establishment.

## Consent

Written informed consent was obtained from the patient for publication of this case report and accompanying images.

## Author contribution

Chaker Kaoutar: Corresponding author writing the paper

Ahmed Brahim Ahmedou : Corresponding author writing the paper

Youssef Oukessou: study concept

Sami Rouadi: study concept

Reda Abada: study concept

Mohamed Roubal : correction of the paper

Mohamed Mahtar : correction of the paper

## Registration of research studies

researchregistry2464

## Guarantor

Dr Ahmed Brahim Ahmedou

Dr Chakir Kaoutar.
